# Awareness and Knowledge of Pharmacists toward Biosimilar Medicines: A Survey in Jordan

**DOI:** 10.1155/2022/8080308

**Published:** 2022-06-27

**Authors:** Muna Oqal, Bushra Hijazi, Abdelrahim Alqudah, Ahmad Al-Smadi, Basima A Almomani, Roaa Alnajjar, Majd Abu Ghunaim, Mohammad Irshaid, Aroob Husam

**Affiliations:** ^1^Department of Pharmaceutics and Pharmaceutical Technology, Faculty of Pharmaceutical Sciences, The Hashemite University, P.O Box 330127, Zarqa 13133, Jordan; ^2^Department of Clinical Pharmacy, Faculty of Pharmacy, Jordan University of Science and Technology, Irbid, Jordan; ^3^Department of Clinical Pharmacy and Pharmacy Practice, Faculty of Pharmaceutical Sciences, The Hashemite University, Zarqa, Jordan; ^4^Department of Adult Health Nursing, Princess Salma Faculty of Nursing, Al Al-Bayt University, Al-Mafraq, Jordan

## Abstract

**Aims:**

Pharmacists in all clinical settings are recognized drug experts and integral educators of biosimilar medicines. Therefore, the objective of this study was to assess pharmacists' knowledge, predictors of knowledge, and views toward biosimilar medicines in Jordan.

**Methods:**

A cross-sectional study was conducted in Jordan during October–December 2020. An Internet-based self-administrated questionnaire on knowledge and views was distributed using social media groups to the pharmacists among different areas in Jordan. A descriptive and univariate analysis was performed. Binary logistic regression was conducted to determine the predictors of knowledge including all variables with *p* < 0.20 on univariate analysis.

**Results:**

A total 536 responses were received, 502 of which were completed (93.7% response rate). A total of 52.6% of the pharmacists were knowledgeable about biosimilar medicines and the mean of knowledge level was 6.47 ± 1.62 (range 2–10). Multivariate analysis identified that respondents who had heard about biosimilars before (OR = 1.942, 95% CI = 1.231–3.063, *p* < 0.05) was more likely to be knowledgeable. Respondents who had not taken the course or the postgraduating training course about biosimilars that were less likely to be knowledgeable (OR = 0.548, 95% CI = 0.357–0.839, *p* < 0.05). A positive response was noted in pharmacist's view regarding the implementation of biosimilar medicines in healthcare setting, biosimilar medicine prescription related to decreased costs, self-study about biosimilar medicine, and incorporating biosimilar education program at the pharmacy school curriculum universities level.

**Conclusions:**

Pharmacists' views and knowledge vary regarding the particularities and key issues on biosimilar medicines in Jordan. Incorporating biosimilar course in pharmacy school curriculum could improve their acceptance for future pharmacy jobs.

## 1. Introduction

Biosimilar is a biological product that has a version of the active substance of an already approved original biological medicine (known as the originator or licensed reference medicine). It is designed to be very similar to their originators in terms of quality, safety, efficacy, immunogenicity, and clinical properties [1–4]. However, the generics of biological medicines are not possible which means making the exact copy by manufacturer is not feasible because biological substances are heterogeneous and complex in nature, high molecular weight, and batch to-batch variability [5]. The approval process of biosimilar medicines is more complex, compared to generic medicines, and it requires extensive investigation to obtain a marketing authorization, including Phase 1 and Phase 3 preclinical studies [6–8]. The regulatory framework for biosimilar medicines is well defined by both European Medicines Agency (EMA) and the Food and Drug Administration (FDA) [2, 4, 6, 9–12]. Biosimilar development costs in comparison to their reference medicines (originator biologic) is low, allowing for potential cost savings of expenditure of pharmaceutical and healthcare systems [1, 4, 13]. Biological and biosimilar medicines have specific pharmacovigilance considerations such as immunogenicity, manufacturing variability, and stability [14, 15]. Therefore, ongoing pharmacovigilance activities should be maintained after approval to ensure their safety, through reporting of adverse reactions [16].

Importantly, pharmacists, being the drug experts, must ensure their accurate understanding of this new category of drugs to assure the safe and optimal use of biosimilars. They must educate themselves and update their knowledge by keeping aware of current medical literature [17]. The biosimilar medicines are becoming available in Jordan, after 3 years of launching in the country where it was first approved [18, 19]. In fact, Jordan has 10 approved biosimilars, the first (erythropoietin) being approved in 2012, in addition to somatropin, human insulin, and a number of mAb biosimilars including two filgrastim, two rituximab, infliximab, trastuzumab, and adalimumab [20]. As biosimilars now are becoming more widely available, pharmacists working in community and hospital settings may need to advise their patients about biosimilars, thus assessing the awareness of pharmacists toward biosimilars is highly important [2]. Several surveys assessing knowledge and awareness, perspectives and attitudes of community, and hospital pharmacists toward biosimilar medicines have been conducted in different countries among the world [2, 12, 21, 22].

However, up to our knowledge no previous studies have been performed before on assessing the knowledge and practice of pharmacists toward biosimilar medicines. Therefore, the main objective of this study was to evaluate the knowledge of pharmacists toward biosimilar medicines and predictors that could influence their knowledge in Jordan. This will help to optimize effective treatment of these new category of drugs to patients. A secondary objective was to explore the views of pharmacists about biosimilar medicines.

## 2. Methods

### 2.1. Design and Data Collection

A cross-sectional design was conducted to meet the study objectives. Data collection was performed between the periods October–December 2020 using an Internet-based self-administrated questionnaire which was created using Google Forms. The participants represented the Jordanian pharmacy workforce which composes of the following: community pharmacy practice which is the most common practice, followed by industry (including sales and marketing), hospital practice, academia and research, regulatory bodies, and others [23] who were recruited through social media platforms such as Jordan Pharmacist Association official social platforms which were used to reach out to all pharmacists in different occupations. The questionnaire was distributed across several Facebook and WhatsApp groups of pharmacists among different areas in Jordan, these social media groups were created as a tool for general communication within the pharmacist's community. Two to three reminders were sent every 2 weeks, and the composition of responses was checked regularly to ensure a representative sample. Data collection was conducted over a period of three months to ensure the collection of a representative sample with adequate size. In addition, informed consent was obtained from the participants as a prerequest to proceed in participation.

### 2.2. Sample Size

The sample size was calculated using Rao soft sample size calculator based on a margin of error of 5%, a 95% confidence level, a population size of 20000, and a response distribution of 50% which will give the largest sample size. The calculated sample size revealed the need for at least was 378 pharmacists. However, for the purpose of enhancing the generalizability of the results, a minimum sample of 500 pharmacists was enrolled in this study.

### 2.3. Ethical Consideration

The study was approved by Institutional Review Board at the Hashemite University in Jordan (Reference number: 2021/2020/4/3).

### 2.4. Development of the Survey Questionnaire

A self-administered questionnaire was created especially for the purpose of this study. Most of the questions related to the knowledge, and views were selected from the literature [12, 24, 25]. Face validity was revised by a group of experts in the field and was constituted of five pharmacologists. The questionnaire is composed of three sections. The first section consisted of eight questions about demographic data including gender, age, area of residence, bachelor's degree (pharmacy or Pharm D), holding a postgraduate degree, professional specialty, length of service in professional specialty, and the university of graduation. In addition, it had a question whether the pharmacists had taken a biosimilar course or postgraduating biosimilar training course. The second section was about the pharmacists' knowledge related to biosimilar medicines which consisted of 10 questions [12]. The respondent pharmacists were asked to indicate whether some statements about biosimilar medicines were accurate or not (yes/no). The total knowledge score ranged from “0” (No knowledge) to “10” (high knowledge). The third section consisted of 13 statements exploring the pharmacists' views about biosimilar medicines [12, 24, 25]. The participating pharmacists were requested to point out their views and agreements about each statement using agree/disagree 5-point Likert scale. The questionnaire was pretested for reliability through the pilot study. The views scale was calculated and showed an excellent reliability with a Cronbach's alpha of 0.885. Piloting of the questionnaire was performed to assess the comprehension and accuracy of the questions in relation to the research topic, identify possible redundancy among the 32 questions, and ensure the usability of the data-collection method.

### 2.5. Data Analysis

Statistical Package for Social Sciences (SPSS) version 24.0 (SPSS Inc., Chicago, IL, USA) was used for analysis of the data. Descriptive statistical analyses were performed to summarize the data for the total sample as counts (percentage). Univariate analysis was performed using a Chi square (*X*^2^) (categorical variables), *t*-test analysis, and One-way ANOVA (continuous variables) as appropriate. A multivariate analysis was conducted to determine predictors of knowledge using binary logistic regression (knowledge) including all variables with *p* < 0.20 on univariate analysis. Statistical significance was set at *p* value <0.05. Odds ratio (OR) values and their 95% confidence intervals (95% CI) were calculated for the predictors of pharmacist's knowledge. Knowledge was dichotomized as knowledgeable and nonknowledgeable. For this purpose, the answers to 10 different questions of knowledge for each participant were labeled as categorical variables using a cut-off point for cumulative scores of correct answers based on the mean of the correct answers of the respondents. A participant was categorized as knowledgeable if the sum of the scores was >6 (out of 10) and nonknowledgeable if the sum of the scores was ≤6 (out of 10).

## 3. Results

### 3.1. Demographics

We received 536 responses of which only 502 responses were completed and included in the analysis, which retains a 93.7% response rate. The demographic characteristics of the participating pharmacists are summarized in Table 1. The majority of respondents (77.9%) was aged between 21 and 30 years, and most of them were female (*n* = 379, 75.5%). More than half of the respondents' area of the residence was from the capital of Jordan, Amman (*n* = 265, 52.8%). The vast majority of participants have a bachelor's degree in pharmacy (*n* = 456, 90.8%), and only 28.7% of the respondent pharmacists have postgraduate certificate. Close to the half of the respondents worked at community pharmacies (46.2%). Most of the respondents (79.1%) have heard about biosimilars before. However, almost the quarter of the respondents (26.1%) had taken a course or a postgraduate training course about biosimilars. More details about the demographics of the respondents are presented in Table 1.

### 3.2. Pharmacists' Knowledge Level about Biosimilar Medicines

Approximately, a half of pharmacists (52.6%, 264/502) were knowledgeable, and the mean number of correct answers was 6.47 ± 1.62 (range 2–10). Only 1% (5/502) of respondents answered all questions correctly and none of the respondents reported knowing nothing at all about biosimilars. The respondents' answers to each of the 10 statements proposed are shown in Table 2. The adequacy of pharmacists' answers to the statements about biosimilar medicines in the questionnaire vary from one statement to another. A minimum percentage of adequate answers obtained was 38.4% (95% CI [34.1–42.7]) for the statement “if biosimilar medicine is structurally identical to its reference medicinal product.” A maximum percentage of adequate answers obtained was up to 76.3% (95% CI, [72.6–80.0]) for the statement “if a drug is for which marketing authorization is granted on the sole investigation of pharmacokinetic bioequivalence with its reference medicinal product.” A detailed comparison between knowledgeable versus nonknowledgeable groups (*n*%, *p*-value) for each knowledge statement is summarized in supplementary Table 1.

### 3.3. Predictors of Pharmacist's Knowledge

As shown in Table 3, the results of univariate analysis indicated that participants' gender, length of service in professional specialty, hearing about biosimilars before, had taken a course or a postgraduate training course about biosimilars were associated with the pharmacist's knowledge with *p* values of <0.20 in the univariate analysis. So these variables were investigated as predictors for knowledge and included in the multivariate analysis. The results of multivariate analysis identified that respondents who had heard about biosimilars before (OR = 1.942, 95% CI = 1.231–3.063, *p*=0.004) were more likely to be knowledgeable. On the other hand, respondents who had not taken course or postgraduate training course about biosimilars were less likely to be knowledgeable (OR = 0.548, 95% CI = 0.357–0.839, *p*=0.006).

### 3.4. Pharmacist's Views about Biosimilar Medicines

The level of pharmacists' views agreement varied from one statement to another as indicated in Table 4. The highest frequency of answers for all statements was supportive (including agree and strongly agree) and ranged from 45.7% to 74.5%. Out of 502 respondents, 41.8% of pharmacists agreed with the implementation of biosimilar medicines in healthcare setting. In addition, 46.8% of pharmacists agreed to trying and testing biosimilar medicines in terms of efficacy and safety. A total of 42.4% of respondents not only agreed that biosimilar medicines are the pharmacist's concern but a 6.6% also strongly disagreed. The pharmacists' responses for the approval substitution of a reference biological medicinal product to its biosimilar product by a pharmacist were between 45.7% (strongly agree/agree) and 32.1% (neutral). More than 60% of pharmacists strongly agreed/agreed that biosimilar medicines prescription allows reducing healthcare costs. The highest percentage of agreement (74.5%) was for the statement, “I prefer to work in a pharmacy that has biosimilar medicines.”

## 4. Discussion

This study has assessed the pharmacists' knowledge level and views about biosimilar medicines in Jordan and to explore the predictors which could influence their knowledge. Few surveys have been conducted to assess pharmacists' knowledge and attitude toward biosimilars in Middle-East countries [26, 27], and up to our knowledge this is the first questionnaire survey that has been conducted in Jordan.

The current study highlighted gaps in biosimilar knowledge and understanding among pharmacists. Our results show that the percentage of community pharmacists participated in this study is approximately half of all participated pharmacists. This might be due to the large number of pharmacists who worked in the community pharmacies compared to those who worked in other pharmacy-related occupations in Jordan [23]. Consistent with the literature, this research found that community pharmacists were less knowledgeable about biosimilar medicines compared to hospital pharmacists [22]. This lack of awareness could be due to the decreased biosimilar prescriptions in outpatient clinics and limited availability of biosimilar medicines in community pharmacies [3, 12]. In agreement with our findings, another study by Pasina et al. indicated low percentage of pharmacists who having complete knowledge about biosimilars [28].

Notably, 61.4% of our respondents lack the knowledge that biosimilar medicine is not structurally identical to its reference medicinal product. This could be explained that similarity between biosimilar medicine and its reference medicinal product does not mean they both have identical structure [1–4]. The FDA defines a biosimilar product as highly similar but not identical to an already licensed biologic product (also termed reference product or biooriginator) in terms of quality, safety, and efficacy [29, 30]. Therefore, it is important to educate pharmacists accurately and promptly by shedding light on some of the confusion differences between biosimilars and their reference biologics [31]. In addition, 61.4% of pharmacists in the present study knew that biosimilar medicine has the same dosage and route of administration compared to its reference medicinal product. This finding could be partly explained by knowing that biosimilars are still considered new drugs and that there is a lack of educational initiatives [2, 3]. In fact, limited reports indicated what specific biosimilar factors contribute to the reluctance and uncertainty of pharmacists to accept biosimilars as equal to the reference product [32–34]. However, awareness of the similarities and differences between reference product and its biosimilar and impact on their efficacy and safety is imperative [35]. Approximately, more than 60% of respondent pharmacists in this study were knowledgeable with the fact that the biosimilar medicine has no meaningful differences from a reference medicinal products in term of quality, safety, and efficacy [36, 37]. Notably, this statement about biosimilar medicines was confirmed by evidence-based information obtained from various clinical trials [38, 39]. Moreover, the most obvious finding to emerge from the analysis is that pharmacists supported the indication extrapolation that refers to the approval of a biosimilar for indications held by the bio-originator but that were not directly evaluated during the biosimilars' clinical trials [3]. This outcome is contrary to that of Adé et al. who found that 64% of pharmacists opposed indication extrapolation as they have doubted biosimilar safety and efficacy in extrapolated indications [24]. This discrepancy could be due to differences in the study populations, the pharmacy curricula, market availability of biosimilars, and the resources of biosimilars among these studies [40, 41].

Data regarding pharmacists' view show that almost two-thirds of our participants (64%) agreed that biosimilar medicines prescription allows for reducing healthcare costs. Noteworthy, the high cost of reference biological medicinal products is the rational for the development of biosimilar medications, as they mitigate rising drug costs in biologics and have significant cost-saving advantages over biological medicinal products [3]. Considering this environment, the availability of biosimilar as alternatives versions of reference biological medicinal products, is critical for containing the healthcare expenses [42–48].

Another important aspect about biosimilar medicine is its interchangeability with reference product. According to definition of biosimilar, an interchangeable biosimilar must be highly similar to reference product and produces the same clinical result in any given patient [3, 49]. It is important to know that the biosimilar substitution policy is not the same among different counties in the world [50, 51]. Our surveyed pharmacists indicated neutral to positive attitudes about interchangeability. These findings match with what was reported by Danese et al. [52, 53]. Notably, interchangeability between biosimilars and reference medicines has numerous debates and are still ongoing because it is associated with a potential to induce immunogenicity, which in turn could affect the efficacy and cause toxicity [1]. Therefore, it is imperative that healthcare professionals who are involved in the use of biosimilar medicines are informed of the considerations related to their prescribing practices, traceability, and interchangeability [54].

Moreover, more than 70% of the respondents believed that pharmacists are the main source of information to educate physicians and patients about the appropriate medication use of these products and the differences between biosimilar and their reference biologics. This finding is expected as the pharmacists are considered the experts in pharmacotherapy, so they play a vital role in evaluating the benefit versus the risk for medications [6, 31]. These results also are in agreement with O'Callaghan et al.'s findings [21]. Additionally, the introduction of biosimilar agents into the Jordan market in 2015 [55], opened the space to pharmacists in Jordan to acquire the necessary knowledge and awareness on the principle aspects surrounding the biosimilar medicines [24].

Furthermore, data show that the majority of respondent pharmacists in favor to educate themselves about biosimilars and encouraged to take courses to enrich their knowledge about biosimilars. In addition, they are in favor with the implementation of biosimilars in healthcare settings. These results corroborate the findings of previous studies in France [12] and Pakistan [56]. Over half of our participants encouraged pharmacy students and pharmacists to take courses in biosimilar medicines to enrich their knowledge and improve their clinical practice which is parallel with other reports [3, 37]. Moreover, our results indicated low percentage of pharmacists (26.1%) who had taken biosimilars a course or a postgraduate training course about biosimilar medicines, albeit, the exact educational activities in our study were not defined, similar to other study [53]. The learning educational means of biosimilar education include training courses, self-study, independent guideline, and/or journal article colleague discussion, continuous education, and consulting promotional manufacturer materials [21, 22, 25, 57–59]. A positive trend toward incorporating biosimilar drug course in pharmacy school curriculum has been noted among respondents. In addition, respondent pharmacists believed that a beneficial impact of biosimilars course at universities would be reflected in improving the acceptance for future pharmacy jobs. These findings are in agreement with a previous study in Karachi [56]. The positive trend of our participants toward working in a pharmacy that has biosimilar medicines is in accord with two studies [53, 60].

Most importantly, our results suggested the knowledge level was significantly positively associated with having heard about biosimilars before or taking a course or postgraduate training course about biosimilars before. These relationships support previous research [58]. Murphy et al. presented results demonstrating a structured biosimilar educational program's ability to improve provider biosimilar confidence [61]. Taken together, biosimilar education not only improves provider understanding and confidence but also elicits actual prescribing changes and increases biosimilar use [3].

### 4.1. Limitation of the Study

One major limitation of this study is the inability to fully track the activity of the online surveys to measure the number of people who opened the survey (number of online hits) and those who completed it (number of people who clicked submit) to compute a response rate. Although the response rate was calculated based on the number of completed out of received responses, we postulate this is maybe lower than the actual response rate since this calculation does not account for pharmacists who only opened the survey but did not click submit. The potential of nonresponse bias as the participants who were not interested in this topic night decline participation compared to others. However, our sample included a representative sample of pharmacists from different specialties, and they were recruited from different geographical areas of Jordan (north, south, and middle). The survey was undertaken during COVID-19 pandemic, and the attitudes of pharmacists may evolve and change over time. It will be valuable to repeat a similar study in different timepoint and then evaluate whether awareness has changed. The detailed information about biosimilar-specific education topics was not obtained. Therefore, further studies of biosimilar-specific education topics are recommended to alleviate existing misunderstandings and bridge knowledge gaps altogether.

## 5. Conclusions

The findings of this study indicate important implications for pharmacist's knowledge regarding key issues of biosimilar medicines in Jordan. This study shows that most pharmacists in Jordan are knowledgeable about biosimilar medicines. In addition, this study highlights the impact of biosimilar education in increasing the familiarity with biosimilar medicines. As well as, it identifies the optimistic willingness of pharmacists in Jordan toward educating themselves about biosimilar and support educational program incorporation in the curriculum of university pharmacy school. That may have a great impact on increasing the awareness and knowledge of pharmacists and other healthcare providers toward biosimilar medicines and their safe and effective use. In addition, they could contribute to strengthen biosimilar market penetration in Jordan market and on the subsequent cost savings. Future research to evaluate the knowledge and views of different healthcare providers (physicians, nurses) and patients in Jordan about biosimilar medicine are encouraged. This has the potential to ensure safe and effective use of biosimilar medicines and to assess perceived biosimilar educational needs.

## Figures and Tables

**Table 1:** Demographic data of pharmacists' respondents (*n* = 502)

Demographic data of pharmacist's respondent (*n* = 502)		
Pharmacist's demographics	Frequency (*n*)	Percentage (%)
*Gender*		
Male	123	24.5
Female	379	75.5

*Age*		
21–30	391	77.9
31–40	76	15.1
Above 40	35	7

*Area of residence*		
Amman	265	52.8
Other than Amman	237	47.2

*BSc degree type*		
Pharmacy	456	90.8
Pharm D	46	9.2

*Postgraduate certificate*		
Bachelor	358	71.3
Postgraduate	144	28.7

*Professional specialty*		
Community pharmacist	232	46.2
Other (medical representative, academia, hospital pharmacist, pharmacologist…)	270	53.8

*Length of service in professional specialty*		
<1 year	200	39.8
1–3 years	138	27.5
4–10 years	106	21.1
>10 years	58	11.6

*Type of graduate university*		
Public	358	71.3
Private	144	28.7

*Hearing about biosimilars before*		
Yes	397	79.1
No	105	20.9

*Taking biosimilars course or postgraduate training course*		
Yes	131	26.1
No	371	73.9

**Table 2:** Pharmacists answers to statements about biosimilar medicines (*n* = 502).

In your opinion, which statements about biosimilar medicines are accurate? a biosimilar medicine:	Adequate answer	Number of adequate answers
		*n* (%)
Is structurally identical to its reference medicinal product	No	139 (38.4)
Is similar to a reference medicinal product that has gone off-patent	Yes	383 (67.3)
Has no meaningful differences from a reference medicinal product in terms of quality	Yes	323 (64.3)
Has no meaningful differences from a reference medicinal product in terms of safety	Yes	359 (71.5)
Has no meaningful differences from a reference medicinal product in terms of efficacy	Yes	348 (69.3)
Has the same dosage and route of administration compared to its reference medicinal product	Yes	194 (38.6)
Is a drug for which marketing authorization is granted on the sole investigation of pharmacokinetic bioequivalence with its reference medicinal product	No	383 (76.3)
Is a drug for which assessment of biosimilarity requires more comprehensive data compared to generic drugs	Yes	386 (76.9)
Requires preclinical and clinical studies	Yes	378 (75.3)
Extrapolation of indications is the authorization of a biosimilar in indications of the reference biologic in the absence of specific clinical trial/data for the biosimilar in those indications	Yes	345 (68.7)

**Table 3:** Univariate and multivariate analysis of factors affecting the pharmacists' knowledge.

All participants	Univariate analysis		Multivariate analysis		
	Not knowledgeable	Knowledgeable	*P* value	Or (95%CI)	*P* value
*Gender*					
Female	186 (78.2)	193 (73.1)	0.189	Ref 1.202 (0.778–1.859)	0.407
Male	52 (21.8), 186 (78.2)	71 (26.9)			

*Age*					
21–30	183 (76.9)	208 (78.8)	0.697		
31–40	36 (15.1)	40 (15.2)			
Above 40	19 (8.0)	16 (6.1)			

*Area of residence*					
Amman	125 (52.5)	140 (53.0)	0.909		
Other than Amman	113 (47.5)	124 (47.0)			

*BSc degree type*					
Pharmacy	219 (92)	237 (89.8)	0.384		
Pharm D	19 (8)	27 (10.2)			

*Professional specialty*					
Community	104 (43.7)	128 (48.5)	0.283		
Pharmacist others	134 (56.3)	136 (51.5)			

*Length of service as in professional specialty*					
>10 years	26 (10.9)	32 (12.1)	0.135	Ref 0.945 (0.510–1.750) 0.64 (0.338–1.213) 1.196 (0.617–2.317)	0.708
<1 year	90 (37.8)	110 (41.7)			
1–3 years	77 (32.4)	61 (23.1)			
4–10 years	45 (18.9)	61 (23.1)			

*Type of graduate university*					
Public	168 (70.6)	190 (72.0)	0.733		
Private	70 (29.4)	74 (28.0)			

*Hearing about biosimilars*					
No	65 (27.3)	40 (15.2)	0.001	Ref 1.942 (1.231–3.063)	0.004
Yes	173 (72.7)	224 (84.8)			

*Biosimilars course or post-graduating training course*					
Yes	46 (19.3)	85 (32.2)	0.001	Ref 0.548 (0.357–0.839)	0.006
No	192 (80.7)	179 (67.8)			

**Table 4:** Pharmacists' level of agreements to statements about biosimilar medicines (*n* = 502).

To what extent do you agree or disagree with the following statements?	Strongly disagree *n* (%)	Disagree *n* (%)	Neutral *n* (%)	Agree *n* (%)	Strongly agree *n* (%)
I am in favor with the implementation of biosimilar medicines in healthcare setting	23 (4.6)	20 (4)	173 (34.5)	210 (41.8)	76 (15.1)
Biosimilar medicines are tried and tested in terms of efficacy and safety	27 (5.4)	19 (3.8)	94 (18.7)	235 (46.8)	127 (25.3)
Biosimilar medicines are not only pharmacist's concern	33 (6.6)	44 (8.8)	116 (23.1)	213 (42.4)	96 (19.1)
I approve the substitution by a pharmacist of a reference biological medicinal product to its biosimilar product	28 (5.6)	84 (16.7)	161 (32.1)	169 (33.7)	60 (12)
Biosimilar medicines prescription allows for reducing healthcare costs	21 (4.2)	34 (6.8)	126 (25.1)	210 (41.8)	111 (22.1)
I intend to educate myself about biosimilar medicines in clinical practice to improve patient safety	170 (33.9)	17 (3.4)	15 (3)	196 (39)	104 (20.7)
I encourage pharmacy students and pharmacists to take courses in biosimilar medicines to enrich their knowledge and improve their clinical practice	188 (37.5)	11 (2.2)	18 (3.6)	198 (39.4)	87 (17.3)
I prefer to work in a pharmacy that has biosimilar medicines	96 (19.1)	11 (2.2)	21 (4.2)	198 (39.4)	176 (35.1)
Pharmacists are the main source of information to educate physicians, other clinicians, and patients about the appropriate medication use of these products and the differences between biosimilar and their reference biologics	156 (31.3)	13 (2.6)	25 (5)	203 (40.4)	105 (20.9)
Biosimilar course may benefit the pharmacy profession	161 (32.1)	10 (2)	13 (2.6)	237 (47.2)	81 (16.1)
Incorporating biosimilar course in pharmacy school curriculum will be important to pharmacist future career	153 (30.5)	15 (3)	12 (2.4)	240 (47.8)	82 (16.3)
Teaching biologics and biosimilar drugs course to undergraduate pharmacy students will be important to patient safety	144 (28.7)	14 (2.8)	12 (2.4)	237 (47.2)	95 (18.9)
Incorporating biologics and biosimilar drugs course in the pharmacy college curriculum is important in improving future pharmacy graduates' acceptance of future jobs	154 (30.7)	14 (2.8)	15 (3)	212 (42.2)	107 (21.3)

## Data Availability

The data that support the findings of this study are available from the corresponding author upon reasonable request.

## Additional Points

Biosimilar is a biological product that has a version of the active substance of an already approved original biological medicine. Several surveys assessing knowledge and awareness, perspectives and attitudes of community and hospital pharmacists toward biosimilar medicines have been conducted in different countries. Most of pharmacists are informed about the biosimilar medicines. However, there is a lack of understanding of the application of that knowledge. What is new. This study identifies some predictors for biosimilar knowledge among participated pharmacists. This research highlights the importance to pledge educational activities for pharmacists about biosimilar medicines to ensure their safe and optimal use which is necessary to control log-term costs of biologics. This study reveals a positive trend toward incorporating biosimilar drug course in pharmacy school curriculum among respondents, as this could be reflected in improving their acceptance for future pharmacy jobs. The findings of this study support the need for conducting further studies of biosimilar-specific education topics to alleviate existing misunderstandings and bridge knowledge gaps altogether about biosimilar medicines among pharmacists.

## Supplementary Materials

Supplementary Materials Supplementary Table 1: Knowledge of Jordanian pharmacist regarding biosimilar medicines. (knowledgeable vs. nonknowledgeable).

## References

[1] Ingrasciotta Y., Cutroneo P. M., Marcianò I., Giezen T., Atzeni F., Trifirò G. (2018). Safety of biologics, including biosimilars: perspectives on current status and future direction. *Drug Safety*.

[2] Pawłowska I., Pawłowski L., Krzyżaniak N., Kocić I. (2019). Perspectives of hospital pharmacists towards biosimilar medicines: a survey of polish pharmacy practice in general hospitals. *BioDrugs*.

[3] Leonard E., Wascovich M., Oskouei S., Gurz P., Carpenter D. (2019). Factors affecting health care provider knowledge and acceptance of biosimilar medicines: a systematic review. *Journal of managed care & specialty pharmacy*.

[4] European Medicines Agency (2014). Guideline on similar biological medicinal products. https://www.ema.europa.eu/en/documents/scientific-guideline/guideline-similar-biological-medicinal-products-rev1_en.pdf.

[5] Aronson J. K., Ferner R. E. (2016). How similar are biosimilars?. *British Medical Journal*.

[6] Lucio S. D., Stevenson J. G., Hoffman J. M. (2013). Biosimilars: implications for health-system pharmacists. *American Journal of Health-System Pharmacy*.

[7] Kurki P., Ekman N. (2015). Biosimilar regulation in the EU. *Expert Review of Clinical Pharmacology*.

[8] Calvo B., Zuñiga L. (2012). The US approach to biosimilars. *BioDrugs*.

[9] European Medicines Agency (2012). Guideline on similar biological medicinal products containing monoclonal antibodies—non-clinical and clinical issues. https://www.ema.europa.eu/en/documents/scientific-guideline/guideline-similar-biological-medicinal-products-containing-monoclonal-antibodies-non-clinical_en.pdf.

[10] Schellekens H., Smolen J. S., Dicato M., Rifkin R. M. (2016). Safety and efficacy of biosimilars in oncology. *The Lancet Oncology*.

[11] Ngo D., Chen J. (2021). A clinical review of biosimilars approved in oncology. *The Annals of Pharmacotherapy*.

[12] Beck M., Michel B., Rybarczyk-Vigouret M.-C. (2017). Knowledge, behaviors and practices of community and hospital pharmacists towards biosimilar medicines: results of a French web-based survey. *Monoclonal Antibodies*.

[13] McCarthy G., Ebel Bitoun C., Guy H. (2013). Introduction of an infliximab biosimilar (ct-P13): a five-year budget impact analysis for the treatment of rheumatoid arthritis in Ireland. *Value in Health*.

[14] European Medicines Agency (2016). Guideline on good pharmacovigilance practices (GVP) product- or population-specific considerations II: biological medicinal products. https://www.ema.europa.eu/en/documents/scientific-guideline/guideline-good-pharmacovigilance-practices-gvp-product-population-specific-considerations-ii_en-0.pdf.

[15] Casadevall N., Edwards I. R., Felix T. (2013). Pharmacovigilance and biosimilars: considerations, needs and challenges. *Expert Opinion on Biological Therapy*.

[16] Gonzalez-Gonzalez C., Lopez-Gonzalez E., Herdeiro M. T., Figueiras A. (2013). Strategies to improve adverse drug reaction reporting: a critical and systematic review. *Drug Safety*.

[17] Cavell G. (2009). Expert pharmacist roles are needed to champion medication safety. https://www.researchgate.net/publication/291636154_Expert_pharmacist_roles_are_needed_to_champion_medication_safety.

[18] Jordan Food and Drug (2016). JFDA administration. https://www.fda.gov/drugs/drug-safety-and-availability/2016-drug-safety-communications.

[19] Haddadin R. D. (2011). Concept of biosimilar products in Jordan. *Biologicals*.

[20] Kang H.-N., Thorpe R., Knezevic I. (2020). The regulatory landscape of biosimilars: WHO efforts and progress made from 2009 to 2019. *Biologicals*.

[21] O’Callaghan J., Bermingham M., Leonard M. (2017). Assessing awareness and attitudes of healthcare professionals on the use of biosimilar medicines: a survey of physicians and pharmacists in Ireland. *Regulatory Toxicology and Pharmacology*.

[22] Beck M., Michel B., Rybarczyk-Vigouret M.-C. (2016). Rheumatologists’ perceptions of biosimilar medicines prescription: findings from a French web-based survey. *BioDrugs*.

[23] Alefan Q., Halboup A. (2016). Pharmacy practice in Jordan. https://www.sciencedirect.com/science/article/pii/B9780128017142000113.

[24] Adé A., Bourdon O., Bussières J.-F. (2017). A survey of pharmacists’ knowledge and views of biosimilars in Quebec and France. *Annales Pharmaceutiques Françaises*.

[25] Cohen H., Beydoun D., Chien D. (2016). Awareness, knowledge, and perceptions of biosimilars among specialty physicians. *Advances in Therapy*.

[26] Almalki Z. S., Iqbal M. S., Alossaimi M. A. (2020). Physicians’ knowledge and awareness about biosimilars in Saudi arabia: what is imperative to know ??. *Journal of Young Pharmacists*.

[27] Farhat F., Othman A., El Karak F., Kattan J. (2016). Review and results of a survey about biosimilars prescription and challenges in the Middle East and North Africa region. *SpringerPlus*.

[28] Pasina L., Casadei G., Nobili A. (2016). A survey among hospital specialists and pharmacists about biosimilars. *European Journal of Internal Medicine*.

[29] U.S. (2021). F. D. A. Biosimilar development, review, and approval 2017. https://www.fda.gov/Drugs/DevelopmentApprovalProcess/HowDrugsareDevelopedandApproved/ApprovalApplications/TherapeuticBiologicApplications/Biosimilars/ucm580429.html.

[30] Barbier L., Simoens S., Soontjens C., Claus B., Vulto A. G., Huys I. (2021). Off-patent biologicals and biosimilars tendering in europe-A proposal towards more sustainable practices. *Pharmaceuticals*.

[31] Jarrett S., Dingermann T. (2015). Biosimilars are here: a hospital pharmacist’s guide to educating health care professionals on biosimilars. *Hospital Pharmacy*.

[32] Moorkens E., Jonker-Exler C., Huys I., Declerck P., Simoens S., Vulto A. G. (2016). Overcoming barriers to the market access of biosimilars in the European union: the case of biosimilar monoclonal antibodies. *Frontiers in Pharmacology*.

[33] Rémuzat C., Dorey J., Cristeau O., Ionescu D., Radière G., Toumi M. (2017). Key drivers for market penetration of biosimilars in Europe. *Journal of Market Access & Health Policy*.

[34] Aapro M. S. (2012). What do prescribers think of biosimilars?. *Targeted Oncology*.

[35] Stevenson J. G. (2015). Clinical data and regulatory issues of biosimilar products. *American Journal of Managed Care*.

[36] Guidance Document (2015). Scientific considerations in demonstrating biosimilarity to a reference product. https://www.fda.gov/regulatory-information/search-fda-guidance-documents/scientific-considerations-demonstrating-biosimilarity-reference-product.

[37] (2016). AMCP partnership forum: biosimilars—ready, set, launch. *Journal of managed care & specialty pharmacy*.

[38] Park W., Hrycaj P., Jeka S. (2013). A randomised, double-blind, multicentre, parallel-group, prospective study comparing the pharmacokinetics, safety, and efficacy of CT-P13 and innovator infliximab in patients with ankylosing spondylitis: the PLANETAS study. *Annals of the Rheumatic Diseases*.

[39] Yoo D. H., Hrycaj P., Miranda P. (2013). A randomised, double-blind, parallel-group study to demonstrate equivalence in efficacy and safety of CT-P13 compared with innovator infliximab when coadministered with methotrexate in patients with active rheumatoid arthritis: the PLANETRA study. *Annals of the Rheumatic Diseases*.

[40] Almohammed O. A., Aldwihi L. A., Alhifany A. A. (2020). Public knowledge, perception, and experience with generic medications in Saudi Arabia. *Saudi Medical Journal*.

[41] Salhia H. O., Ali A., Rezk N. L., El Metwally A. (2015). Perception and attitude of physicians toward local generic medicines in Saudi Arabia: a questionnaire-based study. *Saudi Pharmaceutical Journal*.

[42] Gulácsi L., Brodszky V., Baji P. (2015). Biosimilars for the management of rheumatoid arthritis: economic considerations. *Expert Review of Clinical Immunology*.

[43] Isaacs J. D., Cutolo M., Keystone E. C., Park W., Braun J. (2016). Biosimilars in immune‐mediated inflammatory diseases: initial lessons from the first approved biosimilar anti‐tumour necrosis factor monoclonal antibody. *Journal of Internal Medicine*.

[44] Mestre-Ferrandiz J., Towse A., Berdud M. (2016). Biosimilars: how can payers get long-term savings?. *PharmacoEconomics*.

[45] Brodszky V., Baji P., Balogh O., Péntek M. (2014). Budget impact analysis of biosimilar infliximab (CT-P13) for the treatment of rheumatoid arthritis in six central and Eastern European countries. *The European Journal of Health Economics*.

[46] Jha A., Upton A., Dunlop W. C. N., Akehurst R. (2015). The budget impact of biosimilar infliximab (remsima) for the treatment of autoimmune diseases in five European countries. *Advances in Therapy*.

[47] Beck M., Michel B., Rybarczyk-Vigouret M.-C., Sordet C., Sibilia J., Velten M. (2017). Biosimilar infliximab for the management of rheumatoid arthritis in France: what are the expected savings?. *European Journal of Hospital Pharmacy*.

[48] Farfan-Portet M.-I., Gerkens S., Lepage-Nefkens I., Vinck I., Hulstaert F. (2014). Are biosimilars the next tool to guarantee cost-containment for pharmaceutical expenditures?. *The European Journal of Health Economics*.

[49] Li E., Ramanan S., Green L. (2015). Pharmacist substitution of biological products: issues and considerations. *Journal of Managed Care & Specialty Pharmacy*.

[50] Bocquet F., Paubel P. (2016). First monoclonal antibody biosimilars: tackling the challenge of substitution. *Journal of Medical Economics*.

[51] Vogler S., Schneider P., Zuba M., Busse R., Panteli D. (2021). Policies to encourage the use of biosimilars in European countries and their potential impact on pharmaceutical expenditure. *Frontiers in Pharmacology*.

[52] Park S.-K., Hisamatsu T., Ran Z., Wei S.-C., Park D. I. (2018). Knowledge and viewpoints on biosimilar monoclonal antibodies from members of the Asian Organization of Crohn’s and colitis: comparison with European Crohn’s and Colitis members. *Intestinal research*.

[53] Danese S., Fiorino G., Michetti P. (2016). Changes in biosimilar knowledge among European Crohn’s colitis organization [ECCO] members: an updated survey. *Journal of Crohn’s and Colitis*.

[54] (2015). *Guide to Biosimilars for Healthcare Professionals and Patients*.

[55] (2021). Guideline for registration of biosimilars in Jordan. https://www.jfda.jo/EchoBusV3.0/SystemAssets/PDF/AR/LawsAndRegulation/Drug/RegisterSection/10_303.pdf.

[56] Shakeel S., Hassali M. A., Rehman H., Rehman A. U., Muneswarao J. (2020). Knowledge, attitude, and practice towards biosimilars and interchangeable products: a prescriptive insight by the pharmacists. *International Journal of General Medicine*.

[57] Dylst P., Vulto A., Simoens S. (2014). Barriers to the uptake of biosimilars and possible solutions: a Belgian case study. *PharmacoEconomics*.

[58] van Overbeeke E., De Beleyr B., de Hoon J., Westhovens R., Huys I. (2017). Perception of originator biologics and biosimilars: a survey among Belgian rheumatoid arthritis patients and rheumatologists. *BioDrugs*.

[59] Hallersten A., Fürst W., Mezzasalma R. (2016). Physicians prefer greater detail in the biosimilar label (SmPC)—results of a survey across seven European countries. *Regulatory Toxicology and Pharmacology*.

[60] Kellner H., Domènech’s E., Lakatos’s P. L. (2016). AB0314 awareness and acceptance of biosimilars by rheumatologists in eleven Eu countries. https://www.researchgate.net/publication/310778558_AB0314_Awareness_and_Acceptance_of_Biosimilars_by_Rheumatologists_in_Eleven_Eu_Countries.

[61] Barbier L., Simoens S., Vulto A. G., Huys I. (2020). European stakeholder learnings regarding biosimilars: part II-improving biosimilar use in clinical practice. *BioDrugs*.

