# Supplementary material for: Awareness and Knowledge of Pharmacists toward Biosimilar Medicines: A Survey in Jordan
Source: Int J Clin Pract. 2022 Jun 27;2022:8080308. doi: 10.1155/2022/8080308 (PMC9252697; doi:10.1155/2022/8080308)
Supplement: Supplementary Materials — Supplementary Table 1: Knowledge of Jordanian pharmacist regarding biosimilar medicines. (knowledgeable vs. nonknowledgeable). [file 8080308.f1.docx]

| **In your opinion, which statements about biosimilar medicines are accurate? A biosimilar medicine:** | **Answer** | **Knowledgeable**  **N (%)**  **264 (52.6)** | **Non-knowledgeable**  **N (%)**  **238 (47.4)** | **P-value** |
| --- | --- | --- | --- | --- |
| is structurally identical to its reference medicinal product | - Correct - Incorrect | 124 (47.0)  140 (53.0) | 69 (29.0)  169 (71.0) | 0.000 |
| is similar to a reference medicinal product that has gone off-patent | - Correct - Incorrect | 208 (78.8)  56 (21.2) | 130 (54.6)  108 (45.4) | 0.000 |
| has no meaningful differences from a reference medicinal product in terms of quality | - Correct - Incorrect | 224 (84.8)  40 (15.2) | 99 (41.6)  139 (58.4) | 0.000 |
| has no meaningful differences from a reference medicinal product in terms of safety | - Correct - Incorrect | 235 (89.0)  29 (11.0) | 124 (52.1)  114 (47.9) | 0.000 |
| has no meaningful differences from a reference medicinal product in terms of efficacy | - Correct - Incorrect | 234 (88.6)  30 (11.4) | 114 (47.9)  124 (52.1) | 0.000 |
| has the same dosage and route of administration compared to its reference medicinal product | - Correct - Incorrect | 99 (37.5)  165 (62.5) | 95 (39.9)  143 (60.1) | 0.322 |
| is a drug for which marketing authorization is granted on the sole investigation of pharmacokinetic bioequivalence with its reference medicinal product | - Correct - Incorrect | 232 (87.9)  32 (12.1) | 151 (63.4)  87 (36.6) | 0.000 |
| is a drug for which assessment of biosimilarity requires more comprehensive data compared to generic drugs | - Correct - Incorrect | 242 (91.7)  22 (8.3) | 144 (60.5)  94 (39.5) | 0.000 |
| [requires preclinical and clinical studies | - Correct - Incorrect | 228 (86.4)  36 (13.6) | 150 (63.0)  88 (37.0) | 0.000 |
| extrapolation of indications is the authorization of a biosimilar in indications of the reference biologic in the absence of specific clinical trial/data for the biosimilar in those indications | - Correct - Incorrect | 214 (81.1)  50 (18.9) | 131 (55.0)  107 (45.0) | 0.000 |
